# Predictive potential of somatic symptoms for the identification of subthreshold depression and major depressive disorder in primary care settings

**DOI:** 10.3389/fpsyt.2023.999047

**Published:** 2023-02-14

**Authors:** Xiuwen Li, Huimin Zhang, Xue Han, Lan Guo, Felicia Ceban, Yuhua Liao, Jingman Shi, Wanxin Wang, Yifeng Liu, Weidong Song, Dongjian Zhu, Hongqiong Wang, Lingjiang Li, Beifang Fan, Ciyong Lu, Roger S. McIntyre

**Affiliations:** ^1^Department of Medical Statistics and Epidemiology, School of Public Health, Sun Yat-sen University, Guangzhou, China; ^2^Guangdong Provincial Key Laboratory of Food, Nutrition and Health, Sun Yat-sen University, Guangzhou, China; ^3^Department of Nutrition, Guangdong Engineering Technology Research Center of Nutrition Translation, Guangzhou, China; ^4^Department of Psychiatry, Shenzhen Nanshan Center for Chronic Disease Control, Shenzhen, China; ^5^Mood Disorders Psychopharmacology Unit, University Health Network, Toronto, ON, Canada; ^6^Brain and Cognition Discovery Foundation, Toronto, ON, Canada; ^7^Braxia Health, Mississauga, ON, Canada; ^8^Mental Health Institute of the Second Xiangya Hospital, Central South University, Changsha, China; ^9^Department of Psychiatry, University of Toronto, Toronto, ON, Canada; ^10^Department of Pharmacology, University of Toronto, Toronto, ON, Canada

**Keywords:** subthreshold depression, major depressive disorder, somatic symptoms, primary care, screening, depression

## Abstract

**Background:**

The presence of heterogenous somatic symptoms frequently obscures the recognition of depression in primary care. We aimed to explore the association between somatic symptoms and subthreshold depression (SD) and Major Depressive Disorder (MDD), as well as to determine the predictive potential of somatic symptoms in identifying SD and MDD in primary care.

**Methods:**

Data were derived from the Depression Cohort in China study (ChiCTR registry number: 1900022145). The Patient Health Questionnaire-9 (PHQ-9) was used to assess SD by trained general practitioners (GPs), and the Mini International Neuropsychiatric Interview depression module was used to diagnose MDD by professional psychiatrists. Somatic symptoms were assessed using the 28-item Somatic Symptoms Inventory (SSI).

**Results:**

In total of 4,139 participants aged 18–64 years recruited from 34 primary health care settings were included. The prevalence of all 28 somatic symptoms increased in a dose-dependent manner from non-depressed controls to SD, and to MDD (*P* for trend <0.001). Hierarchical clustering analysis grouped the 28 heterogeneous somatic symptoms into three clusters (Cluster 1: energy-related symptoms, Cluster 2: vegetative symptoms, and Cluster 3: muscle, joint, and central nervous symptoms). Following adjustment for potential confounders and the other two clusters of symptoms, per 1 increase of energy-related symptoms exhibited significant association with SD (*OR* = 1.24, 95% *CI*, 1.18–1.31) and MDD (*OR* = 1.50, 95% *CI*, 1.41–1.60) The predictive performance of energy-related symptoms in identifying individuals with SD (*AUC* = 0.715, 95% *CI*, 0.697–0.732) and MDD (*AUC* = 0.941, 95% *CI*, 0.926–0.963) was superior to the performance of total SSI and the other two clusters (*P* < 0.05).

**Conclusions:**

Somatic symptoms were associated with the presence of SD and MDD. In addition, somatic symptoms, notably those related to energy, showed good predictive potential in identifying SD and MDD in primary care. The clinical implication of the present study is that GPs should consider the closely related somatic symptoms for early recognition for depression in practice.

## Introduction

The diagnosis and treatment of Major Depressive Disorder (MDD) is a preeminent public health challenge due to its high prevalence and extensive disease burden (1). Globally, depressive disorders are ranked as the single greatest contributor to non-fatal health loss (7.5% of all Years Lived with Disability) (1). The total estimated number of individuals living with depression increased by 18.4% between 2005 and 2015 (2), with trends likely to increase in the near future due to factors including but not limited to rapid socio-economic development and increased psychosocial stress. Subthreshold depression (SD, also called minor or subsyndromal depression), defined as depressive symptoms present but not meeting the diagnostic threshold for MDD, is regarded as the precursor for incident major depression (3, 4). Persons with SD are nearly twice as likely to develop major depression compared to non-depressed controls (4). Thus, the early detection and management of SD is critical in reducing the onset and severity of clinical depression.

Primary care is the most frequent entry point into the healthcare system. Accordingly, in most countries, the vast majority of individuals with depression are screened, diagnosed, and treated in primary care (5, 6). Although epidemiologic studies have shown that approximately 20% of patients present to primary care with clinically significant depressive symptoms (7), over 50% of patients with depression remain undiagnosed and untreated (8, 9) due to factors including, but not limited to, inadequate diagnostic skills, limited consultation time, and heterogeneous presentations of depression (10, 11). Among the foregoing factors, somatic symptoms (i.e., headache, back pain, fatigue, and heart palpitations) of depression are a predominant hindrance to the recognition of depression in primary care. Although several reports have shown that emotional and cognitive symptoms are prominent both in SD and MDD (12, 13), patients with depression who go to primary care would like to complain about various somatic symptoms rather than emotional and cognitive symptoms (14, 15), which in turn may lead to misdiagnosis with physical disease (16). In western countries, it is reported that 66–93% of patients with depression exhibit somatic symptoms (17, 18), whereas in China, more than 70% of patients with depression exhibit moderate to severe somatic symptoms (19). In addition, patients may be hesitant to discuss psychological distress in non-psychiatric settings, and instead, choose to focus on their somatic symptoms. Therefore, improving the evaluation of depressive-related somatic symptoms may be feasible and effective toward the early recognition and management of SD and MDD in primary care.

The importance of somatic symptoms in SD and MDD has been raised in the past decades. Tuithof et al. found that chronic physical disorders, which may cause various somatic symptoms, were risk factors for SD to MDD (20). Castellini et al. reported that several somatic symptoms including migraine, headache, and fatigue could serve as early signs of mood disorders (21). Our previous study (13), which aimed to examine the impact of cognitive-affective and somatic domains on the transitions of SD, also found that compared to the remission and intermittent group, participants with persistent depressive symptoms during 12 months showed significantly higher somatic symptom scores. Besides, McMahon and colleagues reported that somatic symptoms in energy level accounted for the elevated prevalence of SD among pregnant women (22). Novick et al. found that among various somatic symptoms, pain-related symptoms showed the greatest prognostic value for treatment response and remission in patients with MDD (23). Thus, somatic symptoms are gaining increasing attention both in SD and MDD, and the results varied in previous studies due to different populations and the heterogeneity of somatic symptoms. However, there is limited research investigating the extensive somatic symptoms across the spectrum from SD to MDD in the general population. Besides, considering the high heterogeneity of various somatic symptoms, exploring the associations between different clusters of somatic symptoms and depression might help to recognize the most closely depressive-related somatic symptoms.

Therefore, the present population-based study was conducted to (1) explore the correlations between extensive somatic symptoms and depressive symptoms, and identify different clusters of somatic symptoms *via* cluster analysis, (2) the association between total somatic symptoms, as well as different clusters of somatic symptoms, and SD and MDD, (3) evaluate whether assessing total and different clusters of somatic symptoms would be useful toward the early identification of SD and MDD in primary care.

## Participants and methods

### Study design and participants

Data were derived from the Depression Cohort in China (DCC) study (ChiCTR registry number: 1900022145), which is an ongoing population-based longitudinal study with the aim to improve early identification, treatment, prevention, and management of subthreshold and clinical depression. The DCC study uses a Toronto-based Building Bridges to Integrate Care (BRIDGES) model (24) to standardize the screening, diagnosis, and treatment of subthreshold depression and depression. A detailed description of the DCC study design has been described elsewhere (25).

In our analysis, participants aged 18–64 years were recruited from 34 primary health care settings between January 2019 and November 2020. Exclusion criteria were: (1) diagnosis of severe psychiatric disorder (i.e., bipolar disorder, schizophrenia, schizoaffective mental disorder, paranoid mental disorder mental disorders caused by epilepsy, or mental retardation), and/or alcohol or drug addiction disorder; (2) pregnant or perinatal women; (3) non-fluency in mandarin; (4) inability to understand study questionnaires or provide informed consent. Additionally, participants were excluded from all analyses if they were missing or had incomplete data concerning: demographic characteristics, smoking, drinking, body mass index (BMI), sleep duration, sleep quality, evaluation of somatic symptoms, and evaluation of depression. All study procedures were carried out in accordance with the Declaration of Helsinki, and written informed consent was obtained from all participants.

### Study measures

#### Diagnosis of subthreshold depression and major depressive disorder

Participants were consecutively recruited and first screened for SD by trained general practitioners (GPs) at the point of enrollment. Participants with SD were further referred to the psychiatry department to take part in the face-to-face Mini-International Neuropsychiatric Interview (MINI) to receive the diagnosis of MDD within 12 months.

SD was assessed using the Patient Health Questionnaire-9 (PHQ-9) administered by trained GPs, and the PHQ-9 was previously identified as the most reliable tool for screening depression (26). SD was operationalized as a total PHQ-9 score ≥5 and no current or past history of MDD. Since higher PHQ-9 scores are associated with a higher risk of MDD, and a cut-off score of ≥10 has the best accuracy to detect MDD (27, 28), participants with SD were divided into low-risk and high-risk groups in the subsequent analysis. The Cronbach α for PHQ-9 was 0.77 in this study.

According to PHQ-9 and MINI assessments, all study participants were divided into 4 groups: (1) the non-depressed control group (*n* = 900) was operationalized as PHQ-9 total score ≤ 4 without a history of MDD; (2) the low-risk group (*n* = 2,044) was operationalized as PHQ-9 total score 5–9 without a history of MDD; (3) the high-risk group (*n* = 975) was operationalized as PHQ-9 ≥ 10 and no definitive clinical diagnosis of MDD according to the MINI; (4) the MDD group (*n* = 220) consisted of individuals with a definitive clinical diagnosis of MDD in accordance with the MINI depression module.

#### Assessment of somatic symptoms

Somatic symptoms were assessed using the 28-item Somatic Symptoms Inventory (SSI), a self-report scale appraising extensive somatic symptoms which have bothered the respondent in the past week. The SSI rates the respondent's degree of discomfort for each of the included 28 symptoms from 1 to 5 (1 = absent; 2 = a little bit; 3 = moderate; 4 = quite a bit; 5 = a great deal). The total SSI score (i.e., the sum of all items) was used to quantify the severity of somatic symptoms. The Cronbach α for PHQ-9 was 0.XXX in this study. The Cronbach α for SSI was 0.95 in this study.

#### Additional covariates

Additional covariates, including demographic characteristics, health status, and behavioral habits, were assessed *via* self-report questionnaires. Chronic disease was defined as the prevalence of any of the following: (1) hypertension; (2) diabetes; (3) heart disease; (4) apoplexy; (5) thyroid disease; (6) dyslipidemia; (7) hyperuricemia; (8) gastrointestinal issues (i.e., chronic gastritis, gastric ulcer, gastroesophageal reflux); (9) history of tumors. Body mass index (BMI) was calculated as weight in kilograms divided by height in meters squared. Sleep duration was defined as self-reported actual sleep time at night, and sleep quality was self-assessed across 5 levels ranging from very good to very poor.

### Statistical analysis

Continuous variables were reported as the median (interquartile range, IQR), and compared using the Kruskal–Wallis H test for overall groups. Categorical variables were expressed by measures of frequency and percentages, and intergroup comparisons were analyzed *via* the chi-squared test. The percent prevalence of every somatic symptom (self-reporting any of the following options: a little bit; moderate; quite a bit and a great deal) was calculated separately according to different groups. The Cochran-Armitage trend test was used to determine the trend in the prevalence of each somatic symptom, and the percentage of individuals exhibiting a mean SSI item score (i.e., total SSI score divided by 28) ≥2 from non-depressed controls to SD, and to MDD.

Bivariate correlations between each PHQ-9 and SSI item were determined *via* Kendall correlation coefficient analysis. Hierarchical clustering analysis was employed to yield clusters of 28 SSI items (based on their Kendall correlation coefficients matrix with PHQ-9 items), and the number of clusters was determined based on the proportion of variation in the data captured by the clusters. To validate the accuracy and stability of the clustering, participants were randomly divided into a discovery set and a validation set at a 1:1 ratio in hierarchical clustering analysis, and external criteria of Rand index (29) of the clusters derived from the two independent datasets were calculated to obtain numerical comparison values.

Subsequently, a multivariable-adjusted general linear model (GLM) was used to evaluate the linear trend of different clusters of somatic symptoms from non-depressed controls to SD, and to MDD. To estimate the odds ratios (ORs) per 1 score increase of total SSI or different clusters for SD, and MDD, multinomial logistic regression analysis was performed rather than an ordinal regression model because the parallel regression assumption was violated. Receiver operating characteristic (ROC) curve analysis was performed to test the predictive potential of total SSI or different clusters of somatic symptoms in identifying participants with SD or MDD, using the bootstrap method to compare different measures of the area under the curve (AUC). All statistical analyses were performed using R (4.2.1). A 2-sided *P* < 0.05 was considered statistically significant.

## Results

### Characteristics of study population

A total of 4,139 participants were included in this study. The median (IQR) age for all the participants was 36.0 (29.0, 47.0) years and 37.7% were male. Among all participants, 900 (21.7%) were classified into the non-depressed control group, 3,019 (73.0%) exhibited SD, of which 2,044 (49.4%) were stratified to the low-risk group and 975 (23.6%) to the high-risk group. A total of 220 (5.3%) participants were definitively diagnosed with MDD. Baseline characteristics, including demographic factors, health status, behavioral habits, PHQ-9 score, and SSI score, are summarized in Table 1. Median age and frequency of smoking in the past month were balanced among participants within different groups. Compared with the normal group, participants with SD or MDD had higher rates of single status or divorce, chronic diseases, increased frequency of drinking alcohol in the past month, lower BMI, lower exercise frequency, shorter sleep duration, and worse sleep quality.

**Table 1:** Baseline characteristics of the study participants.

	**Overall** **(*n =* 4,139)**	**Non-depressed** **(*n =* 900)**	**SD**		**MDD** **(*n* = 220)**	***P* value**
			**Low risk** **(*****n*** = **2,044)**	**High risk** **(*****n*** = **975)**		
**Demographic characteristics**						
**Age, y**	36.0 (29.0, 47.0)	37.0 (30.0, 46.0)	36.0 (29.0, 48.0)	36.0 (28.0, 48.0)	38.0 (29.0, 49.0)	0.348
**Gender**, ***N*** **(%)**						< 0.001
Male	1,560 (37.7)	347 (38.6)	832 (40.7)	317 (32.5)	64 (29.1)	
Female	2,579 (62.3)	553 (61.4)	1,212 (59.3)	658 (67.5)	156 (70.9)	
**Marital status**, ***N*** **(%)**						< 0.001
Single	1,007 (24.3)	177 (19.7)	495 (24.2)	274 (28.1)	61 (27.7)	
Married	2,977 (71.9)	706 (78.4)	1,478 (72.3)	655 (67.2)	138 (62.7)	
Divorce	127 (3.1)	14 (1.6)	58 (2.8)	37 (3.8)	18 (8.2)	
Widowed	28 (0.7)	3 (0.3)	13 (0.6)	9 (0.9)	3 (1.4)	
**Education (%)**						< 0.001
Below college	1,578 (38.1)	286 (31.8)	776 (38.0)	442 (45.3)	74 (33.6)	
College or above	2,561 (61.9)	614 (68.2)	1,268 (62.0)	533 (54.7)	146 (66.4)	
**Employed status**, ***N*** **(%)**						< 0.001
Employed	2,596 (62.7)	601 (66.8)	1,307 (63.9)	574 (58.9)	114 (51.8)	
Self-employed	603 (14.6)	124 (13.8)	319 (15.6)	134 (13.7)	26 (11.8)	
Retired	347 (8.4)	64 (7.1)	160 (7.8)	92 (9.4)	31 (14.1)	
Un-employed	593 (14.3)	111 (12.3)	258 (12.6)	175 (17.9)	49 (22.3)	
**Family income**, ***N*** **(%)**						0.001
No fixed income	385 (9.3)	83 (9.2)	178 (8.7)	106 (10.9)	18 (8.2)	
< 5,000¥	470 (11.4)	112 (12.4)	198 (9.7)	129 (13.2)	31 (14.1)	
5,000–9,999¥	1,063 (25.7)	204 (22.7)	525 (25.7)	273 (28.0)	61 (27.7)	
10,000–19,999¥	1,213 (29.3)	247 (27.4)	625 (30.6)	279 (28.6)	62 (28.2)	
20,000–49,999¥	768 (18.6)	193 (21.4)	398 (19.5)	140 (14.4)	37 (16.8)	
50,000–79,999¥	129 (3.1)	28 (3.1)	69 (3.4)	26 (2.7)	6 (2.7)	
>80,000¥	111 (2.7)	33 (3.7)	51 (2.5)	22 (2.3)	5 (2.3)	
**Living status** ***N*** **(%)**						0.001
Single	493 (11.9)	80 (8.9)	257 (12.6)	117 (12.0)	39 (17.7)	
With relatives	3,071 (74.2)	728 (80.9)	1,520 (74.4)	681 (69.8)	142 (64.5)	
With non-relatives	575 (13.9)	92 (10.2)	267 (13.1)	177 (18.2)	39 (17.7)	
**Health status and behavioral habits**						
**Chronic disease**, ***N*** **(%)**						0.001
No	3,395 (82.0)	746 (82.9)	1,692 (82.8)	799 (81.9)	158 (71.8)	
Yes	744 (18.0)	154 (17.1)	352 (17.2)	176 (18.1)	62 (28.2)	
**Frequency for smoking in the past month**, ***N*** **(%)**						0.188
No smoking	3,036 (73.4)	675 (75.0)	1,503 (73.5)	697 (71.5)	161 (73.2)	
5 days or below	633 (15.3)	139 (15.4)	302 (14.8)	152 (15.6)	40 (18.2)	
6 days or above	470 (11.4)	86 (9.6)	239 (11.7)	126 (12.9)	19 (8.6)	
**Frequency for drinking in the past month**, ***N*** **(%)**						0.004
No drinking	1,673 (40.4)	338 (37.6)	879 (43.0)	388 (39.8)	68 (30.9)	
5 days or below	2,009 (48.5)	450 (50.0)	949 (46.4)	482 (49.4)	128 (58.2)	
6 days or above	457 (11.0)	112 (12.4)	216 (10.6)	105 (10.8)	24 (10.9)	
**BMI, kg/m** ^**2**^	22.0 (20.0, 24.1)	22.2 (20.3, 24.3)	22.0 (20.0, 24.2)	21.6 (19.5, 23.9)	21.8 (19.8, 23.8)	< 0.001
**Exercise habit per week (at least 1 time and** **≥30 min)**, ***N*** **(%)**						< 0.001
No	2,374 (57.4)	452 (50.2)	1,137 (55.6)	655 (67.2)	130 (59.1)	
Yes	1,765 (42.6)	448 (49.8)	907 (44.4)	320 (32.8)	90 (40.9)	
**Sleep duration, h**	7.0 (6.0, 7.0)	7.0 (6.0, 8.0)	7.0 (6.0, 7.0)	6.0 (5.0, 7.0)	6.0 (5.0, 7.0)	< 0.001
**Sleep quality**, ***N*** **(%)**						< 0.001
Very good	287 (6.9)	145 (16.1)	124 (6.1)	16 (1.6)	2 (0.9)	
Good	1,133 (27.4)	375 (41.7)	627 (30.7)	111 (11.4)	20 (9.1)	
Average	1,607 (38.8)	319 (35.4)	910 (44.5)	328 (33.6)	50 (22.7)	
Poor	842 (20.3)	57 (6.3)	333 (16.3)	359 (36.8)	93 (42.3)	
Very poor	270 (6.5)	4 (0.4)	50 (2.4)	161 (16.5)	55 (25.0)	
**PHQ-9 score and SSI score**						
**PHQ-9 score**	6.0 (5.0, 10.0)	2.0 (0.0, 3.0)	6.0 (5.0, 7.0)	12.0 (11.0, 16.0)	16.0 (12.0, 20.0)	< 0.001
Somatic	3.0 (2.0, 5.0)	1.0 (0.0, 2.0)	3.0 (3.00, 4.0)	7.0 (5.0, 8.0)	8.0 (6.0, 10.0)	< 0.001
Cognitive	3.0 (2.0, 5.0)	0.0 (0.0, 1.0)	3.0 (2.0, 4.0)	6.0 (5.0, 8.0)	9.0 (6.0, 11.0)	< 0.001
**SSI score**	36.0 (31.0, 45.0)	31.0 (29.0, 36.0)	34.0 (31.0, 41.0)	45.0 (36.0, 59.0)	57.0 (45.0, 73.2)	< 0.001

Values were expressed as median (interquartile range), frequency, and percentage (%). The Kruskal–Wallis H test for quantitative variables and the chi-square test for categorical variables were performed. SD, subthreshold depression; MDD, major depressive disorder; BMI, body mass index; PHQ-9, patient health questionnaire-9; SSI, somatic symptoms inventory. The Non-depressed control group was defined as PHQ-9 total score ≤ 4 without a history of MDD; the low-risk group was defined as PHQ-9 total score 5–9 without a history of MDD; the high-risk group was defined as PHQ-9 ≥ 10 and no definitive clinical diagnosis of MDD; the MDD group consisted of individuals with a definitive clinical diagnosis of current or recurrent MDD in accordance with the MINI depression module.

### Prevalence of somatic symptoms across participant groups

The prevalence of all 28 somatic symptoms assessed by the SSI (with presence operationalized as an item score ≥ 2) was higher in participants with SD or MDD compared to the non-depressed control group. Furthermore, individuals with MDD demonstrated a higher prevalence of all 28 somatic symptoms compared to individuals with SD. The results of the Cochran-Armitage trend test demonstrated that the prevalence of all 28 somatic symptoms increased when going from the non-depressed control group to SD, and then to MDD (*P* for trend < 0.001) (Table 2). Similarly, the prevalence of a mean item score (i.e., total SSI score divided by 28) ≥2 increased going from the non-depressed control group to SD, and then to MDD (*P* for trend < 0.001). The prevalence of several symptoms, including feeling faint or dizzy, feeling not in as good physical health as most friends, feeling weak in parts of the body, and not feeling well most of the time in the past few years, was less than 25% in the non-depressed control group, compared to >50% in the high-risk group, and >75% in persons with MDD.

**Table 2:** Prevalence (%) of Somatic symptoms in different groups.

	**Non-depressed** **(*n =* 900)**	**SD**		**MDD** **(*n* = 220)**	***P* for trend**
**SSI symptoms**		**Low risk** **(*****n*** = **2,044)**	**High risk** **(*****n*** = **975)**		
01. Nausea and vomiting	6.2	14.8	34.9	36.8	< 0.001
02. Muscles soreness	36.0	48.3	61.9	73.2	< 0.001
03. Pains or cramps in your abdomen	10.7	20.1	35.5	42.3	< 0.001
04. Feeling faint or dizzy	23.6	42.2	68.2	83.2	< 0.001
05. Trouble with your vision	34.1	38.4	55.9	65.0	< 0.001
06. Muscles twitching or jumping	9.7	17.8	31.9	44.5	< 0.001
07. Feeling fatigued, weak, or tired all over	40.2	63.6	84.6	92.3	< 0.001
08. A fullness in your head or nose	19.3	28.7	50.3	71.4	< 0.001
09. Pain in your lower back	16.7	25.7	43.6	51.8	< 0.001
10. Constipation	18.2	26.3	39.3	43.2	< 0.001
11. Trouble catching your breath	3.2	8.3	28.0	44.1	< 0.001
12. Hot or cold spells	6.9	11.9	34.2	43.2	< 0.001
13. A ringing or buzzing in your ears	11.9	17.8	35.0	44.5	< 0.001
14. Pains in your heart or chest	6.1	12.3	29.5	46.4	< 0.001
15. Difficulty keeping your balance while walking	3.2	7.2	20.2	27.3	< 0.001
16. Indigestion, upset stomach, or acid stomach	17.2	31.9	49.7	53.2	< 0.001
17. The feeling that you are not in as good physical health as most of your friends	22.3	37.8	68.5	84.1	< 0.001
18. Numbness, tingling, or burning in parts on your body	12.6	20.8	37.9	52.3	< 0.001
19. Headaches	19.1	31.2	54.9	67.3	< 0.001
20. A lump in your throat	11.2	17.8	35.0	47.7	< 0.001
21. Feeling weak in parts of your body	17.2	29.2	58.2	76.8	< 0.001
22. Not feeling well most of the time in the past few years	10.9	23.1	53.6	75.5	< 0.001
23. Heavy feelings in your arms or legs	12.6	24.4	53.3	71.8	< 0.001
24. Your heart pounding, turning over or missing a beat	9.2	15.8	41.3	61.8	< 0.001
25. Your hands and feet not feeling warm enough	9.9	17.3	37.0	50.0	< 0.001
26. The sense that your hearing is not as good as it used to be	12.1	20.1	40.4	54.1	< 0.001
27. Joint pain	17.1	28.4	41.4	49.1	< 0.001
28. Neck pain	27.0	38.6	57.2	68.2	< 0.001
Prevalence of the mean SSI item score ≥2	1.2	6.5	30.1	56.0	< 0.001

The Cochran-Armitage trend test was used to test the trend for the prevalence of each somatic symptom, and the prevalence of the mean SSI item score (i.e., total SSI score divided by 28) ≥ 2 from non-depressed individuals to SD and to MDD. SD, subthreshold depression; MDD, major depressive disorder; SSI, somatic symptoms inventory.

### Correlation analysis and clustering analysis of SSI and PHQ-9

**Figure 1:**
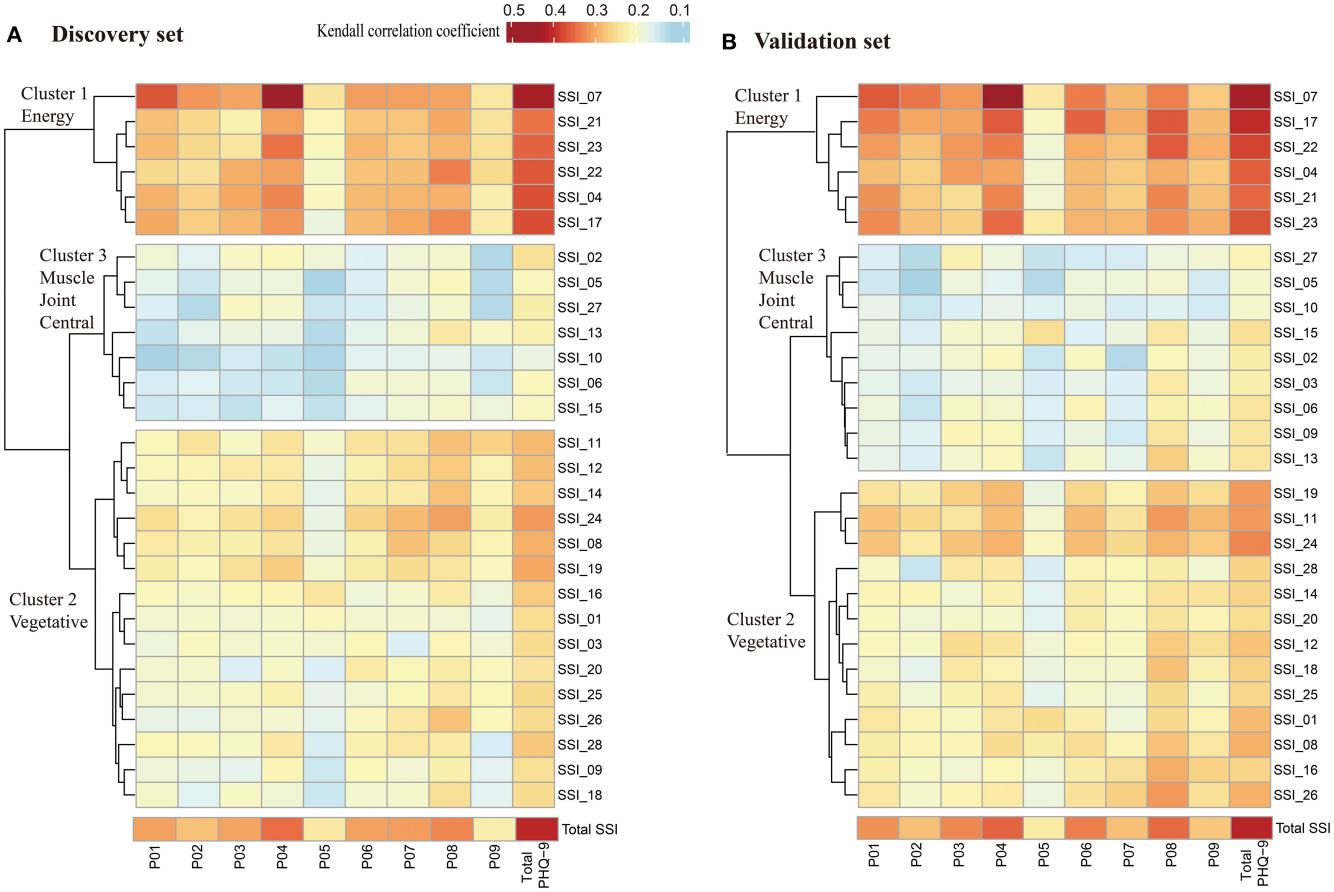
Heatmap of Kendall correlation coefficient between SSI and PHQ-9. **(A)** Heatmap of Kendall correlation coefficient between SSI and PHQ-9 in discovery set (*n* = 2,070); **(B)** Heatmap of Kendall correlation coefficient between SSI and PHQ-9 in validation set (*n* = 2069). Significant correlations were found between each SSI item and each PHQ-9 item using Kendall correlation analysis (*P* < 0.05). Hierarchical clustering analysis was applied to the yield clusters of the 28 SSI items based on their Kendall correlation coefficients with PHQ-9, and the number of clusters was decided based on the proportion of variation in the data captured by the clusters. PHQ-9, indicates patient health questionnaire-9; P01 to P09, represents item 1 to item 9 in PHQ-9; SSI indicates somatic symptoms inventory; SSI_01 to SSI_28, represents item 1 to item 28 in SSI. Cluster 1 consists of 6 energy-related symptoms, including fatigue, weakness, faintness or dizziness, heavy arms or legs, feeling unwell most of the time in the past few years, feeling not in as good physical health most friends; Cluster 2 consists of 15 vegetative symptoms, including nausea and vomiting, pains, or cramps in abdomen, indigestion, upset stomach, or acid stomach, fullness in head or nose, back pain, trouble in catching, breath, pains in heart or chest, heart pounding, turning over or missing a beat, numbness, tingling or burning, headaches, lump in throat, hands and feet not feeling warm enough, sense that hearing is not as good as it used to be; Cluster 3 consists of 7 muscle, joint and central nervous symptoms, including muscles soreness, muscles twitching or jumping, joint pain, trouble with vision, ringing, or buzzing in ears, difficulty in keeping balance while walking, constipation.

We performed Kendall correlation analysis to investigate the correlations between items on the SSI and PHQ-9. A significant correlation was determined between each SSI and PHQ-9 item (*P* < 0.05). A heatmap of the Kendall correlation coefficients is shown in Figure 1. Subsequently, Hierarchical clustering analysis was applied to the yield clusters of unique SSI items based on their Kendall correlation coefficients matrix with each PHQ-9 item. To validate the accuracy and stability of the clustering, participants were randomly divided into a discovery set (*n* = 2,070) and a validation set (*n* = 2,069) at a 1:1 ratio. Three clusters of different somatic symptoms were identified by this unsupervised classification approach in each dataset. In sum, 26 (92.9%) of the 28 SSI items were allocated to the same cluster using the discovery set and validation set with a Rand index of 0.90, indicating good accuracy and stability of the clustering. In both datasets, Cluster 1 consists of 6 energy-related symptoms (SSI items: 4, 7, 17, 21, 22, and 23); Cluster 2 of the discovery set consists of 15 vegetative symptoms (SSI items: 1, 3, 8, 9,11, 12, 14, 16, 18, 19, 20, 24, 25, 26, and 28) and Cluster 3 of the discovery set consists of 7 muscle, joint and central nervous symptoms (SSI items: 2, 5,6, 10, 13, 15, and 27). There were 2 symptoms, SSI item 3 (pains or cramps in your abdomen), and item 9 (pain in lower back) which were grouped in Cluster 2 using the discovery set, but grouped in Cluster 3 using the validation set. Finally, Clusters derived from the discovery set were used in the following analysis according to experts' opinions. The cluster scores were calculated by summing the items in each cluster.

**Figure 2:**
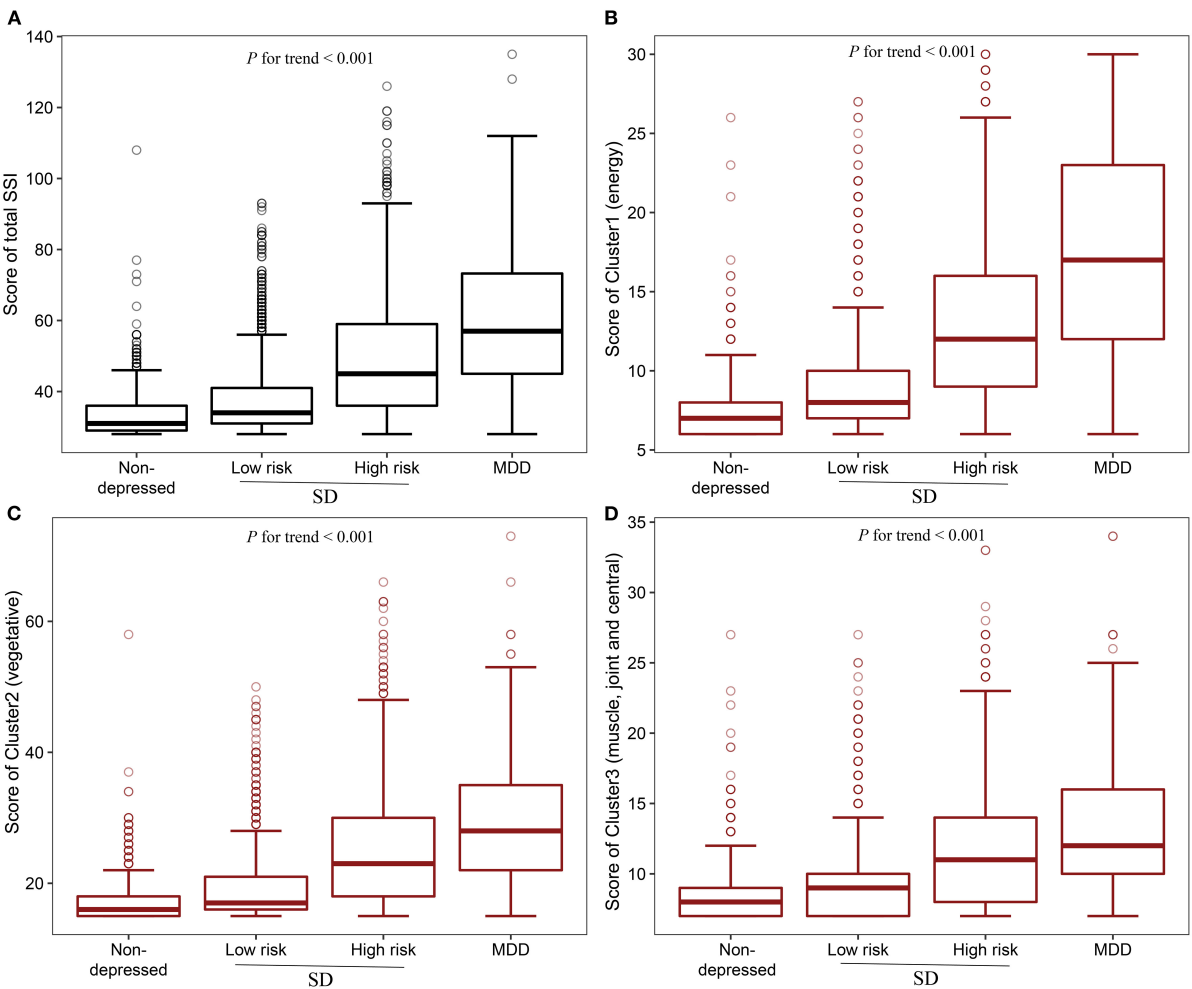
Boxplot of Total SSI score and different clusters scores. **(A)** Total SSI score in different groups; **(B)** Cluster 1(energy-related symptoms) score in different groups; **(C)** Cluster 2 (vegetative symptoms) score in different groups; **(D)** Cluster 3 (muscle, joint and central nervous symptoms) score in different groups. *P* for trend was determined by General linear model (GLM) evaluating the linear trend of the different clusters of somatic symptoms from non-depressed individuals to SD, and to MDD after adjustment for age, gender, marital status, education, employed status, family income, living status, chronic disease, smoking frequency, drinking frequency, BMI, exercise frequency, sleep duration, and sleep quality. SD, subthreshold depression; MDD, major depressive disorder; SSI, somatic symptoms inventory.

The median (IQR) Cluster 1 (energy-related symptoms) scores for non-depressed control, low-risk, high-risk, and MDD groups were 7 (6, 8), 8 (7, 10), 12 (9, 16), and 17 (22, 23), respectively. The median (IQR) Cluster 2 (vegetative symptoms) scores for non-depressed control, low-risk, high-risk, and MDD groups were 16 (15, 18), 17 (16, 21), 23 (18, 30), and 28 (22, 35), respectively. The median (IQR) Cluster 3 (muscle, joint and central nervous symptoms) scores for non-depressed control, low-risk, high-risk, and MDD groups were 8 (7, 9), 9 (7, 10), 11 (8, 14), and 12 (10, 16), respectively (Figure 2). There were significant increasing trends for all three clusters' scores going from non-depressed control to SD and to MDD after adjustment for age, gender, marital status, education, employed status, family income, living status, chronic disease, smoking frequency, drinking frequency, BMI, exercise frequency, sleep duration, and sleep quality (*P* for trend < 0.001).

### Association between total SSI score or cluster scores and presence of SD or MDD

Univariate multinomial logistic regression analysis revealed that, compared to the non-depressed controls, an increase in total SSI score, or an increase in any of the three cluster scores, was associated with an increased odds ratio of SD (both low-risk and high-risk group) and MDD respectively. After controlling for potential confounders, compared to the non-depressed control group, the *OR* for SD and MDD was 1.08 (95% *CI*, 1.07–1.09) and 1.13 (95% *CI*, 1.11–1.14) with per 1 total SSI score increase, respectively (Table 3). When the 28 SSI items were divided into three clusters of somatic symptoms via Hierarchical clustering analysis, after controlling for potential confounders, a per 1 score increase in Cluster 1(energy-related symptoms) was associated with a significant increase in the odds ratio of SD (*OR* = 1.30, 95% *CI*, 1.25–1.35) and MDD (*OR* = 1.53, 95% *CI*, 1.46–1.61) compared to the non-depressed control group, respectively. Moreover, the odds ratio in the high-risk group was greater than in the low-risk group. The increase in the odds ratio of SD and MDD by Cluster 2 (vegetative symptoms) and Cluster 3 (muscle, joint and central nervous symptoms) scores were lower than by Cluster 1 (energy-related symptoms) after adjustment for confounders (Table 3). Even further adjusted for the other two clusters of somatic symptoms, Cluster 1 (energy-related symptoms) was positively associated with the presence of SD (*OR* = 1.24, 95% *CI*, 1.18–1.31) and MDD (*OR* = 1.50, 95% *CI*, 1.41–1.60).

**Table 3:** Association between total SSI score or different cluster scores and the presence of SD or MDD.

	**Unadjusted *OR* (95% *CI*)**	**Model 1 *OR* (95% *CI*)**	**Model 2 *OR* (95% *CI*)**
**Total SSI**			
**Non–depressed**	1.00 (Reference)	1.00 (Reference)	——
**SD**	1.10 (1.09–1.11)	1.08 (1.07–1.09)	——
Low risk	1.08 (1.06–1.09)	1.07 (1.05–1.08)	——
High risk	1.15 (1.14–1.17)	1.13 (1.11–1.14)	——
**MDD**	1.16 (1.14–1.17)	1.13 (1.11–1.14)	——
**Cluster1 (energy)**			
**Non–depressed**	1.00 (Reference)	1.00 (Reference)	1.00 (Reference)
**SD**	1.37 (1.32–1.42)	1.30 (1.25–1.35)	1.24 (1.18–1.31)
Low risk	1.28 (1.23–1.33)	1.23 (1.18–1.29)	1.19 (1.13–1.26)
High risk	1.62 (1.55–1.69)	1.50 (1.43–1.56)	1.42 (1.33–1.51)
**MDD**	1.66 (1.59–1.74)	1.53 (1.46–1.61)	1.50 (1.41–1.60)
**Cluster2 (vegetative)**			
**Non-depressed**	1.00 (Reference)	1.00 (Reference)	1.00 (Reference)
**SD**	1.18 (1.16–1.21)	1.15 (1.12–1.18)	1.06 (1.02–1.10)
Low risk	1.14 (1.11–1.17)	1.12 (1.09–1.15)	1.05 (1.00–1.08)
High risk	1.29 (1.25–1.32)	1.24 (1.20–1.27)	1.10 (1.06–1.15)
**MDD**	1.34 (1.30–1.38)	1.28 (1.24–1.32)	1.06 (1.02–1.11)
**Cluster3 (muscle, joint, and central)**			
**Non-depressed**	1.00 (Reference)	1.00 (Reference)	1.00 (Reference)
**SD**	1.24 (1.20–1.28)	1.18 (1.14–1.23)	0.96 (0.91–1.01)
Low risk	1.16 (1.12–1.21)	1.14 (1.10–1.18)	0.97 (0.92–1.03)
High risk	1.39 (1.34–1.44)	1.30 (1.25–1.36)	0.94 (0.89–1.00)
**MDD**	1.44 (1.38–1.51)	1.33 (1.27–1.39)	0.93 (0.88–1.00)

Model 1, adjusted by age, gender, marital status, education, employed status, family income, living status, chronic disease, smoking frequency, drinking frequency, BMI, exercise frequency, sleep duration, and sleep quality, with only one of total SSI, cluster1, cluster2, or cluster3 score in the model.

Model 2, adjusted by the same covariates in Model 1, with all three of cluster1, cluster2, or cluster3 score in the model.

SD indicates subthreshold depression; MDD, major depressive disorder; SSI, Somatic Symptoms Inventory; *OR*, odds ratio; *CI*, confidence interval.

We also estimate the odds ratio per 1 score increase of total SSI or different clusters in MDD vs. SD subjects, with SD (or its subgroups) as the reference groups (Supplementary Table 1). Compared to the SD group, a per 1 score increase in total SSI and Cluster 1 (energy-related symptoms) was associated with a significant increase in the odds ratio of MDD, respectively. Besides, a per 1 score increase in total SSI and Cluster 1 (energy-related symptoms) was significantly associated with an increase in the odds ratio of MDD, both using the low-risk or high-risk group as the reference group, respectively.

### Predictive potential of total SSI score or cluster scores in identifying SD or MDD

The total SSI score and all three cluster scores demonstrated a moderate but significant ability to identify participants with SD. The *AUC* for total SSI, Cluster 1 (energy-related symptoms), Cluster 2 (vegetative symptoms), and Cluster 3 (muscle, joint and central nervous symptoms) were 0.707 (95% *CI*, 0.689–0.725), 0.715 (95% *CI*, 0.697–0.732), 0.688 (95% *CI*, 0.670–0.706), and 0.641 (95% *CI*, 0.451–0.763), respectively. The sensitivity and specificity were 59.0 and 71.1% for the total SSI score, 66.7 and 64.6% for Cluster 1 (energy-related symptoms), 58.5 and 69.4% for Cluster 2 (vegetative symptoms), and 45.1 and 76.3% for the Cluster 3 (muscle, joint and central nervous symptoms). The predictive potential of the total SSI score in identifying SD was equal to that of Cluster 1 (energy-related symptoms) (*P* for bootstrap method = 0.109), but superior to Cluster 2 (vegetative symptoms) and Cluster 3 (muscle, joint and central nervous symptoms) (*P* for bootstrap method < 0.001). The foregoing results are presented in Table 4.

**Table 4:** Receiver operating characteristic analysis of total SSI score and different Clusters scores in identifying SD and MDD.

	**Cut–off value**	***AUC* (95%*CI*)**	**Sensitivity**	**Specificity**	***P* value**
**Non-depressed vs. SD**					
Total SSI	34.5	0.707 (0.689–0.725)	0.590	0.711	—
Cluster1 (energy)	7.5	0.715 (0.697–0.732)	0.667	0.646	0.109
Cluster2 (vegetative)	17.5	0.688 (0.670–0.706)	0.585	0.694	< 0.001
Cluster3 (muscle, joint, and central)	9.5	0.641 (0.622–0.660)	0.451	0.763	< 0.001
**Non-depressed vs. Low risk**					
Total SSI	33.5	0.647 (0.625–0.668)	0.544	0.662	—
Cluster1 (energy)	7.5	0.649 (0.629–0.670)	0.578	0.646	0.657
Cluster2 (vegetative)	16.5	0.631 (0.610–0.652)	0.620	0.570	0.001
Cluster3 (muscle, joint, and central)	9.5	0.594 (0.572–0.615)	0.373	0.763	< 0.001
**Non-depressed vs. High risk**					
Total SSI	36.5	0.833 (0.815–0.851)	0.738	0.783	—
Cluster1 (energy)	9.5	0.851 (0.834–0.868)	0.695	0.873	< 0.001
Cluster2 (vegetative)	19.5	0.806 (0.787–0.826)	0.653	0.827	< 0.001
Cluster3 (muscle, joint, and central)	9.5	0.740 (0.718–0.761)	0.613	0.763	< 0.001
**Non-depressed vs. MDD**					
Total SSI	41.5	0.932 (0.912–0.951)	0.827	0.893	—
Cluster1 (energy)	10.5	0.941 (0.926–0.963)	0.832	0.917	0.011
Cluster2 (vegetative)	21.5	0.906 (0.881–0.932)	0.782	0.899	< 0.001
Cluster3 (muscle, joint, and central)	9.5	0.836 (0.804–0.867)	0.777	0.763	< 0.001

*P*-value was calculated by the bootstrap method to compare the *AUC* between each Cluster and the total SSI scores.

In distinguishing participants with MDD from the non-depressed control group, Cluster 1 (energy-related symptoms) demonstrated good predictive potential with an *AUC*, sensitivity, and specificity of 0.941 (95% *CI*, 0.926–0.963), 83.2%, and 91.7%, respectively, which was significantly better than the performance of total SSI score (*AUC*, 0.932; 95% *CI*, 0.912–0.951; sensitivity, 82.7%; specificity, 89.3%, *P* for bootstrap method = 0.011). The performance of both Cluster 2 (vegetative symptoms) and Cluster 3 (muscle, joint and central nervous symptoms) in distinguishing MDD were lower than for Cluster 1 (energy-related symptoms) (*P* for bootstrap method < 0.001).

Furthermore, compared with the SD group, participants in the MDD group could be identified by total SSI score with a sensitivity of 80.9% and a specificity of 67.0% (*AUC*, 0.802; 95% *CI*, 0.773–0.831), which was inferior to the performance of the of Cluster 1(energy-related symptoms) (*AUC*, 0. 822; 95% *CI*, 0.793–0.850; sensitivity, 69.5%; specificity, 82.7%, *P* for bootstrap method = 0.004). The ROC analysis results of the total SSI score and different clusters in other subgroup comparisons were concluded in Table 4 and Supplementary Table 2.

We also performed analyses to examine the ROC when a specific cluster was excluded, respectively. The results were concluded in Supplementary Table 3. In distinguishing participants with SD from the non-depressed control group, after excluding Cluster 1 (energy-related symptoms) from the total SSI, the AUC decreased from 0.707 (0.689–0.725) to 0.683 (0.665–0.702), *P* for bootstrap method < 0.001; while after excluding Cluster 3 (muscle, joint and central nervous symptoms) from the total SSI, the AUC increased from 0.707 (0.689–0.725) to 0.716 (0.698-0.734), *P* for bootstrap method < 0.001. In distinguishing participants with MDD from the non-depressed control group, excluding cluster 1 (energy-related symptoms) from the total SSI resulted in a decrease of the AUC from 0.932 (0.912–0.951) to 0.902 (0.876–0.928), *P* for bootstrap method < 0.001; while excluding cluster 3 (muscle, joint and central nervous symptoms) from the total SSI resulted in an increase of the AUC from 0.932 (0.912–0.951) to 0.938 (0.919–0.957), *P* for bootstrap method = 0.022.

## Discussion

In this large population-based study analyzing data derived from primary care settings, we determined that somatic symptoms were correlated with depressive symptoms, and significantly increased in a dose-dependent manner from non-depressed controls to SD, and to MDD. Among all the somatic symptoms assessed by the 28-item SSI, a cluster of energy-related symptoms (including fatigue, weakness, faintness or dizziness, heavy arms or legs, or feeling unwell most of the time in the past few years, feeling not in as good physical health most friends) showed the best performance to identify participants with SD and MDD from non-depressed participants followed by vegetative symptoms. Besides, the ability of energy-related symptoms to discern SD and MDD was superior to that of the total SSI score. To our knowledge, this is the first large population-based study to investigate the effects of somatic symptoms across the spectrum from non-depressed controls to SD and to MDD, and to evaluate the predictive ability of total SSI score and different clusters of somatic symptoms in identifying SD and MDD.

The identification of depression-related somatic symptoms might be feasible and effective for the recognition of depression. However, there is currently no gold standard method to assess for somatic symptoms associated with depression in primary care. In addition, a standard classification for the heterogeneous and abundant array of somatic symptoms does not currently exist. The 28-item SSI, which includes 28 different somatic symptoms across the entire body, is always used to assess somatic symptoms in patients with depression and other psychological disorders (30, 31). Previous studies have subdivided the 28 SSI items into different dimensions to explore the associations between different dimensions and the clinical outcomes of diagnosed MDD (19, 23, 32–34). Compared with the previous studies mentioned above, our study focused on a spectrum of participants including SD and MDD, and aimed to evaluate whether a particular cluster of depression-related somatic symptoms could demonstrate the greatest screening potential for SD and MDD. In light of the different study populations and study objectives, we did not believe the aforementioned classification of somatic symptoms to be the optimal somatic symptom grouping strategy for screening depression. *Via* our unsupervised classification approach, we determined three clusters (energy, vegetative, and other) of somatic symptoms, which were different from the two-dimension (painful and non-painful) or four-dimension (pain, autonomic, energy, and central nervous symptoms) classification in the previous studies (19, 23, 33). Somatic symptoms are heterogeneous that may include different biological entities and require different management. The total SSI included extensive somatic symptoms which could be interpreted as the total burden of physical symptoms, while the three clusters yielded by clustering analysis represent different domains. The previous studies determined that pain symptoms but not the other dimensions were closely associated with the clinical outcomes of diagnosed MDD (23, 32, 33). Interestingly, in our study, the energy cluster demonstrated superior predictive performance in identifying SD and MDD compared to vegetative and other somatic symptoms. Our results indicated that energy-related somatic symptoms may be more useful for screening depression in primary care, followed by vegetative symptoms. The other symptoms, including muscle, joint and central nervous symptoms, might be less useful in such areas.

Although many studies have shown that emotional and cognitive symptoms are prominent in SD (35) and MDD (12), patients with depression who go to primary care would like to complain about various somatic symptoms rather than emotional and cognitive symptoms (14, 15). As a result, a growing body of evidence supported that somatic symptoms are predominant hindrances to the recognition of depression in primary care (14–16). In contrast, recent research held the opinion that somatic symptoms might represent a valid tool for early recognition of depression for the first request of help to GPs when enough attention was paid to the depression-related somatic symptoms (21). In accordance with the Castellini et al. study (21), our results supported that somatic symptoms could be interpreted as an early sign of depression, and represent a valid indication for the GPs diagnostic process of depression. Our previous study (13), which aimed to explore the impact factor on the longitudinal illness deterioration in subjects with SD, found that cognitive-affective symptoms in SD are at greater risk of illness deterioration. At the same time, we also found that compared to subjects showing a remission or intermittent trend, subjects with persistent SD during 12 months showed higher baseline and followed-up somatic symptom scores (13), which indicated that higher burden of somatic symptoms could also play negative effects on worse SD transition. In the current study aiming at examining the predictive potential of somatic symptoms for the early identification of SD and MDD at the point of screening, we further found that somatic symptoms, especially the energy, and vegetative symptoms, showed good predictive potential in identifying SD and MDD in primary care settings. Given that most countries do not recommend screening depression routinely using tools including the emotional and cognitive symptoms in primary care (5), as well as the high initiative complaint of various somatic symptoms in patients with depression who seek help from the GPs (14, 15), it is important to improve the awareness of depression-related somatic symptoms both to GPs and patients at the screening at point of care.

Because the recruitment and assessment period of some participants occurred during the COVID-19 pandemic, concerns about the pandemic and related health measurements might have impacts on the somatic and depressive symptoms of participants. The prevalence of somatic symptoms in our study were higher than that in previous study (21). Nonetheless, consistent with the previous studies before the COVID-19 pandemic (21), our study found that somatic symptoms could help to early recognize depression in primary care. The significance of somatic symptoms in SD and MDD has been raised in the past decades. However, there is limited literature concerning the extensive somatic symptoms across the spectrum of depression. Besides, the ability of the heterogeneous somatic symptoms to identify SD and MDD was not clear. Our study extends the foregoing knowledge base in the following aspects. First, somatic symptoms were highly present in SD compared to non-depressed persons, and increased in a dose-dependent manner from non-depressed individuals to SD and then to MDD in the community population. Second, the extensive somatic symptoms were positively correlated with depressive symptoms, and different clusters of depression-related somatic symptoms could be drawn according to the closeness of correlation. Third, somatic symptoms showed good ability in distinguishing SD and MDD, especially the energy-related symptoms. Taken together, somatic symptoms should be investigated thoroughly in the management of the full range of depression. As the idea of preventing depression gains more traction (36), the assessment and treatment of SD have become a priority (37–39). Further studies to elucidate whether treating somatic symptoms in SD could help to reduce incident MDD are warranted.

The results of our study should be interpreted within the context of several limitations. First, the study participants were only recruited from primary care settings in China, therefore our findings may not generalize to other countries due to factors including but not limited to differences in cultural practices and healthcare systems. However, it is noteworthy that literature from other countries has recognized the importance of somatic symptoms in identifying MDD. Second, the somatic symptoms in our study were assessed *via* the 28-item SSI; we did not measure and analyze other somatic symptoms which may be present in our study population. We plan to expand to include additional somatic symptoms in future studies. Third, participants in our study were recruited from 34 primary health care settings who seek help for basic medical services, and GPs selectively screened those who had mental health-related physical complaints (e.g., sleep problems and chronic somatic pain) or are more likely to have mental health issues based on the GPs' clinical experience and our study training (25). However, the information about the treatment and resolution of specific symptoms leading to the consultation was not collected, which might have a potential influence on the psychic wellbeing of participants. Fourth, information on family history of psychiatric disorders other than depression was not collected in our study. Despite these limitations, to our knowledge, this is the first large population study conducted in primary care settings to investigate the association of somatic symptoms with SD and MDD, and to evaluate whether somatic symptoms are useful toward the recognition of SD and MDD in primary care settings. The strengths of this study included the large representative community-based sample, and the use of a clinically validated diagnostic interview (i.e., MINI) to diagnose MDD.

## Conclusion

Herein, we established that somatic symptoms were associated with the presence of SD and MDD, and increased in a dose-dependent manner from non-depressed controls to SD, and to MDD. Moreover, somatic symptoms showed good predictive performance in identifying SD and MDD in primary care settings. Besides, a cluster of energy-related symptoms showed the best identifying ability followed by vegetative symptoms. The clinical implication of the present study is that GPs should consider the closely related somatic symptoms for early recognition and management of depression in practice.

## Data availability statement

The raw data supporting the conclusions of this article will be made available by the authors, without undue reservation.

## Ethics statement

The studies involving human participants were reviewed and approved by Sun Yat-sen University School of Public Health Institutional Review Board. The patients/participants provided their written informed consent to participate in this study.

## Author contributions

XL, LG, BF, and CL conceived and designed the study. XL performed data analysis and drafted the manuscript. HZ acquired, analyzed, and interpreted the data. YLia, JS, WW, YLiu, WS, DZ, and HW collected the data. HZ, YLia, and XH supervised the study in the 34 primary care settings. LG, LL, FC, RM, BF, and CL provided guidance to revise the draft. All authors critically revised the manuscript for scientific content and approved the final version of the article.
