# Supplementary material for: Predictive potential of somatic symptoms for the identification of subthreshold depression and major depressive disorder in primary care settings
Source: Front Psychiatry. 2023 Feb 14;14:999047. doi: 10.3389/fpsyt.2023.999047 (PMC9971499; doi:10.3389/fpsyt.2023.999047)
Supplement: Supplementary file 1 [file Table_1.docx]

**Supplement**

**Supplementary Table 1. ORs of per 1 total SSI score or different cluster scores increase** **in MDD vs. SD subjects**

|  | **Unadjusted**  ***OR* (95% *CI*)** | **Model 1**  ***OR* (95% *CI*)** | **Model 2**  ***OR* (95% *CI*)** |
| --- | --- | --- | --- |
| **SD vs. MDD** | | | |
| Total SSI | 1.05(1.05-1.06) | 1.04(1.04-1.05) | --- |
| Cluster1 (energy) | 1.21(1.19-1.24) | 1.18(1.15-1.22) | 1.21(1.16-1.26) |
| Cluster2 (vegetative) | 1.09(1.08-1.10) | 1.07(1.06-1.19) | 1.00(0.98-1.03) |
| Cluster3 (muscle, joint and central) | 1.17(1.14-1.20) | 1.12(1.09-1.16) | 0.95(0.90-1.01) |
| **Low risk vs. MDD** | | | |
| Total SSI | 1.10(1.09-1.11) | 1.09(1.08-1.10) | --- |
| Cluster1 (energy) | 1.38(1.33-1.43) | 1.36(1.30-1.41) | 1.33(1.26-1.41) |
| Cluster2 (vegetative) | 1.18(1.16-1.20) | 1.16(1.14-1.19) | 1.06(1.02-1.11) |
| Cluster3 (muscle, joint and central) | 1.29(1.24-1.34) | 1.24(1.19-1.29) | 0.91(0.86-0.97) |
| **High risk vs. MDD** | | | |
| Total SSI | 1.03(1.02-1.03) | 1.03(1.02-1.03) | --- |
| Cluster1 (energy) | 1.12(1.09-1.15) | 1.11(1.18-1.14) | 1.14(1.10-1.19) |
| Cluster2 (vegetative) | 1.04(1.03-1.06) | 1.04(1.02-1.15) | 0.99(0.96-1.02) |
| Cluster3 (muscle, joint and central) | 1.08(1.05-1.11) | 1.07(1.14-1.10) | 0.98(0.93-1.03) |
| **Low risk vs. High risk** | | | |
| Total SSI | 1.07(1.06-1.08) | 1.06(1.05-1.06) | --- |
| Cluster1 (energy) | 1.26(1.23-1.29) | 1.21(1.18-1.24) | 1.19(1.15-1.23) |
| Cluster2 (vegetative) | 1.13(1.11-1.14) | 1.10(1.18-1.12) | 1.05(1.03-1.08) |
| Cluster3 (muscle, joint and central) | 1.19(1.16-1.22) | 1.14(1.11-1.17) | 0.94(0.91-0.97) |

Model 1, adjusted by age, gender, marital status, education, employed status, family income, living status, chronic disease, smoking frequency, drinking frequency, BMI, exercise frequency, sleep duration, and sleep quality, with only one of total SSI, cluster1, cluster2, or cluster3 score in the model.

Model 2, adjusted by the same covariates in Model 1, with all three of cluster1, cluster2, or cluster3 score in the model.

Cluster 1 consists of 6 energy-related symptoms, including fatigue, weakness, faintness or dizziness, heavy arms or legs, feeling unwell most of the time in the past few years, feeling not in as good physical health most friends; Cluster 2 consists of 15 vegetative symptoms, including nausea and vomiting, pains or cramps in abdomen, indigestion, upset stomach, or acid stomach, fullness in head or nose, back pain, trouble in catching, breath, pains in heart or chest, heart pounding, turning over or missing a beat, numbness, tingling or burning, headaches, lump in throat, hands and feet not feeling warm enough, sense that hearing is not as good as it used to be; Cluster 3 consists of 7 muscle, joint and central nervous symptoms, including muscles soreness, muscles twitching or jumping, joint pain, trouble with vision, ringing or buzzing in ears, difficulty in keeping balance while walking, constipation.

SD indicates subthreshold depression; MDD, major depressive disorder; SSI, Somatic Symptoms Inventory; *OR*, odds ratio; *CI*, confidence interval.

**Supplementary Table 2.** **Receiver operating characteristic analysis of total SSI score and different clusters scores in MDD vs. SD subjects.**

|  | **Cut-off value** | ***AUC* (95%*CI*)** | **Sensitivity** | **Specificity** | ***P* value** |
| --- | --- | --- | --- | --- | --- |
| **SD vs MDD** |  |  |  |  |  |
| Total SSI | 42.5 | 0.802(0.773-0.831) | 0.809 | 0.670 | --- |
| Cluster1 (energy) | 13.5 | 0.822(0.793-0.850) | 0.695 | 0.827 | 0.004 |
| Cluster2 (vegetative) | 21.5 | 0.778(0.746-0.809) | 0.782 | 0.678 | <0.001 |
| Cluster3 (muscle, joint and central) | 11.5 | 0.719(0.684-0.754) | 0.595 | 0.750 | <0.001 |
| Low risk vs MDD |  |  |  |  |  |
| Total SSI | 42.5 | 0.868(0.841-0.894) | 0.809 | 0.788 | --- |
| Cluster1 (energy) | 11.5 | 0.884(0.858-0.910) | 0.791 | 0.858 | 0.016 |
| Cluster2 (vegetative) | 21.5 | 0.841(0.812-0.871) | 0.782 | 0.786 | <0.001 |
| Cluster3 (muscle, joint and central) | 11.5 | 0.770(0.735-0.804) | 0.595 | 823 | <0.001 |
| High risk vs MDD |  |  |  |  |  |
| Total SSI | 45.5 | 0.665(0.626-0.703) | 0.741 | 0.508 | --- |
| Cluster1 (energy) | 13.5 | 0.691(0.652-0.730) | 0.695 | 0.623 | 0.003 |
| Cluster2 (vegetative) | 21.5 | 0.644(0.605-0.682) | 0.782 | 0.453 | <0.001 |
| Cluster3 (muscle, joint and central) | 11.5 | 0.613(0.573-0.653) | 0.595 | 0.596 | <0.001 |

*P*-value was calculated by the bootstrap method to compare the *AUC* between each cluster and the total SSI scores.

Cluster 1 consists of 6 energy-related symptoms, including fatigue, weakness, faintness or dizziness, heavy arms or legs, feeling unwell most of the time in the past few years, feeling not in as good physical health most friends; Cluster 2 consists of 15 vegetative symptoms, including nausea and vomiting, pains or cramps in abdomen, indigestion, upset stomach, or acid stomach, fullness in head or nose, back pain, trouble in catching, breath, pains in heart or chest, heart pounding, turning over or missing a beat, numbness, tingling or burning, headaches, lump in throat, hands and feet not feeling warm enough, sense that hearing is not as good as it used to be; Cluster 3 consists of 7 muscle, joint and central nervous symptoms, including muscles soreness, muscles twitching or jumping, joint pain, trouble with vision, ringing or buzzing in ears, difficulty in keeping balance while walking, constipation.

SD indicates subthreshold depression; MDD, major depressive disorder; SSI, somatic symptoms inventory; ROC, receiver operating characteristic curve; *AUC*, area under the curve; *CI*, confidence interval.

The Non-depressed control group was defined as PHQ-9 total score ≤ 4 without a history of MDD; the low-risk group was defined as PHQ-9 total score 5-9 without a history of MDD; the high-risk group was defined as PHQ-9≥10 and no definitive clinical diagnosis of MDD; the MDD group consisted of individuals with a definitive clinical diagnosis of current or recurrent MDD in accordance with the MINI depression module.

**Supplementary Table 3. Receiver operating characteristic of SSI score before and after exclusion of specific clusters**

|  | **Cut-off value** | ***AUC* (95%*CI*)** | **Sensitivity** | **Specificity** | ***P* value** |
| --- | --- | --- | --- | --- | --- |
| **Non-depressed vs SD** | | | | | |
| Total SSI | 34.5 | 0.707(0.689-0.725) | 0.590 | 0.711 | --- |
| Cluster1 excluded | 26.5 | 0.683(0.665-0.702) | 0.568 | 0.698 | <0.001 |
| Cluster2 excluded | 17.5 | 0.701(0.683-0.720) | 0.546 | 0.751 | 0.073 |
| Cluster3 excluded | 25.5 | 0.716(0.698-0.734) | 0.605 | 0.709 | <0.001 |
| Non-depressed vs Low risk | | | | | |
| Total SSI | 33.5 | 0.647(0.625-0.668) | 0.544 | 0.662 | --- |
| Cluster1 excluded | 24.5 | 0.628(0.606-0.649) | 0.638 | 0.526 | <0.001 |
| Cluster2 excluded | 17.5 | 0.640(0.619-0.661) | 0.440 | 0.751 | 0.062 |
| Cluster3 excluded | 22.5 | 0.655(0.634-0.677) | 0.782 | 0.439 | 0.004 |
| Non-depressed vs High risk | | | | | |
| Total SSI | 36.5 | 0.833(0.815-0.851) | 0.738 | 0.783 | --- |
| Cluster1 excluded | 28.5 | 0.800(0.780-0.820) | 0.681 | 0.796 | <0.001 |
| Cluster2 excluded | 18.5 | 0.831(0.812-0.849) | 0.722 | 0.812 | 0.433 |
| Cluster3 excluded | 27.5 | 0.843(0.825-0.861) | 0.737 | 0.804 | <0.001 |
| **Non-depressed vs MDD** | | | | | |
| total SSI | 41.5 | 0.932(0.912-0.951) | 0.827 | 0.893 | --- |
| Cluster1 excluded | 30.5 | 0.902(0.876-0.928) | 0.814 | 0.853 | <0.001 |
| Cluster2 excluded | 19.5 | 0.929(0.909-0.949) | 0.873 | 0.861 | 0.520 |
| Cluster3 excluded | 32.5 | 0.938(0.919-0.957) | 0.818 | 0.929 | 0.022 |

*P*-value was calculated by the bootstrap method to compare the *AUC* before and after exclusion of specific clusters.

Cluster 1 consists of 6 energy-related symptoms, including fatigue, weakness, faintness or dizziness, heavy arms or legs, feeling unwell most of the time in the past few years, feeling not in as good physical health most friends; Cluster 2 consists of 15 vegetative symptoms, including nausea and vomiting, pains or cramps in abdomen, indigestion, upset stomach, or acid stomach, fullness in head or nose, back pain, trouble in catching, breath, pains in heart or chest, heart pounding, turning over or missing a beat, numbness, tingling or burning, headaches, lump in throat, hands and feet not feeling warm enough, sense that hearing is not as good as it used to be; Cluster 3 consists of 7 muscle, joint and central nervous symptoms, including muscles soreness, muscles twitching or jumping, joint pain, trouble with vision, ringing or buzzing in ears, difficulty in keeping balance while walking, constipation.

SD indicates subthreshold depression; MDD, major depressive disorder; SSI, somatic symptoms inventory; ROC, receiver operating characteristic curve; *AUC*, area under the curve; *CI*, confidence interval.

The Non-depressed control group was defined as PHQ-9 total score ≤ 4 without a history of MDD; the low-risk group was defined as PHQ-9 total score 5-9 without a history of MDD; the high-risk group was defined as PHQ-9≥10 and no definitive clinical diagnosis of MDD; the MDD group consisted of individuals with a definitive clinical diagnosis of current or recurrent MDD in accordance with the MINI depression module.
